# Sexually Harassing Behaviors from Patients or Clients and Care Workers’ Mental Health: Development and Validation of a Measure

**DOI:** 10.3390/ijerph17072570

**Published:** 2020-04-09

**Authors:** Sylvie Vincent-Höper, Mareike Adler, Maie Stein, Claudia Vaupel, Albert Nienhaus

**Affiliations:** 1 Department of Work and Organizational Psychology, Universität Hamburg, 20146 Hamburg, Germany; maie.stein@uni-hamburg.de; 2 Department of Occupational Medicine, Hazardous Substances and Public Health, Institution for Statutory Accident Insurance and Prevention in the Healthcare and Welfare Services, 22089 Hamburg, Germany; Mareike.Adler@bgw-online.de (M.A.); Claudia.Vaupel@bgw-online.de (C.V.); Albert.Nienhaus@bgw-online.de (A.N.); 3 Institute for Health Services Research in Dermatology and Nursing, University Clinic Hamburg-Eppendorf, 20246 Hamburg, Germany

**Keywords:** healthcare, measurement, mental health, sexual harassment, validation

## Abstract

Although evidence reveals severe effects of sexual harassment on care workers’ mental health, there is a scarcity of studies that investigate care workers’ experiences of sexually inappropriate behavior from patients or clients. One reason for this lack of research is that validated measures that assess different types of sexual harassment experienced by employees working with patients or clients are lacking. In this study, we seek to establish a conceptual framework for investigating extraorganizational sexual harassment in healthcare work. Based on this theoretical framework, we developed and validated a measure for assessing sexually harassing behaviors from patients or clients. Data were gathered from heterogeneous samples of employees working in a variety of settings in healthcare. To evaluate the factorial structure of the measure, we conducted exploratory factor analysis (EFA) using a calibration sample (*N* = 179) and confirmatory factor analysis (CFA) using a cross-validation sample (*N* = 305). The construct validity of the measure was demonstrated by investigating relationships with indicators of care workers’ mental health. EFA revealed three factors, namely, nonverbal, verbal, and physical acts of sexual harassment. Examination of the measure comprising 14 items revealed acceptable internal consistencies and substantial correlations with indicators of care workers’ mental health. This study provides a useful and sound measure for assessing sexual harassment from patients or clients and paves the way for the development of a comprehensive theoretical framework for the assessment of sexual harassment. Furthermore, it facilitates future investigations of risk factors for sexual harassment and protective factors helping healthcare workers cope with sexual harassment from patients or clients.

## 1. Introduction

Although sexual harassment in the workplace is a relevant and prevailing topic [1], research in this field is fragmented and fraught with problems. A major challenge is that meaningful prevalence rates are lacking, which may be due to the many and varied definitions of sexual harassment and the different methods applied (e.g., varying time periods in which exposure to sexual harassment is measured and single-item measures) [2]. Moreover, it is noted that theoretical developments are weak and that construct validity issues are rarely considered [1,3,4,5]. 

However, the severe negative effects of sexual harassment highlight the need for more rigorous research. Sexual harassment was not only identified as one of the most damaging and ubiquitous barriers to career success and satisfaction but it was also shown to have detrimental effects on employees’ physical and mental health [6,7]. A meta-analysis on the antecedents and consequences of sexual harassment at work revealed a significant impact on physical and mental ill health and even symptoms of posttraumatic stress disorder [6]. 

A representative survey of 1531 employees in Germany showed that, in more than half of the incidents of sexual harassment, customers or clients were the perpetrators. Furthermore, relative to other industries, healthcare and social services workers reported the highest rates of sexual harassment [8]. Although these findings indicate that sexual harassment from individuals outside the organization is a prevalent issue in healthcare work, much of the existing research addressed sexual harassment from colleagues and supervisors [1] while neglecting sexual harassment from clients or patients [9,10,11]. In an effort to compare different types of occupations, a study among more than 1000 organizations in Denmark revealed that care workers encounter sexual harassment from individuals outside the organization (e.g., customers, clients, and patients) most often [12]. Although empirical studies are scarce, some evidence indicates that sexual harassment from patients or clients has severe negative consequences for employees’ mental and physical health [2,13]. However, the effect of sexual harassment from patients or clients on employees’ mental health is yet to be expanded into conceptual approaches and measures. Because research focused on sexual harassment from colleagues and supervisors, measures for assessing this type of sexual harassment exist, such as the Sexual Experiences Questionnaire (SEQ) [3], whereas validated measures for assessing sexual harassment from patients or clients are missing.

To yield in-depth insights into an extraorganizational perspective on sexual harassment, we need a psychometrically sound and valid measure. To understand sexual harassment in healthcare work, efforts must be made to recognize a more comprehensive picture of the sexual harassment experienced by care workers, including sexual harassment from patients or clients. Therefore, the objective of this article is to develop and validate a measure for assessing sexual harassment from patients or clients in healthcare. This article aims to contribute to the existing literature in two ways. Firstly, we aim to advance the examination of sexual harassment from patients or clients and further the understanding of the effects of sexual harassment from patients or clients on care workers’ mental health. Secondly, we seek to make a case for elaborating a theoretical framework underlying sexual harassment from patients or clients in healthcare work. We believe that a validated measure that allows the development of a comprehensive theoretical framework may contribute to future research on sexual harassment from patients or clients.

### 1.1. Conceptualization of Sexual Harassment

After 40 years of research, the definition of sexual harassment is still a controversial issue, and a broadly accepted definition does not exist [1,9,14]. According to Cortina and Berdahl’s [9] review of the sexual harassment literature, sexual harassment can be viewed from three different perspectives: legal, social–psychological, and public/lay. In this study, we focus on the social–psychological perspective to investigate its role in the context of employees’ psychological well-being. Although a generally accepted definition is missing, the different definitions agree that sexually inappropriate behavior is unwanted and causes harm to the victim. In this study, we use a definition of sexual harassment that emphasizes the victim’s subjective interpretation and individual emotional reactions to the experienced sex-related behaviors. Accordingly, sexual harassment is defined as “unwanted sex-related behavior at work that is appraised by the recipient as offensive, exceeding her resources, or threatening her [or his] well-being” [14]. To further refine the conceptualization of sexual harassment, we rely on guidelines of the European Commission of the European Union (EU) [15], which emphasize that sexual harassment comprises a range of inappropriate behaviors, including three types of harassment: nonverbal, verbal, and physical sexual harassment.

Nielsen and colleagues [4] stated that care workers are often exposed to sexual harassment from patients and that research shows that such exposure may have detrimental effects on care workers’ mental health. At the same time, the authors pointed out that studies focusing on care workers’ experiences of sexual harassment from patients or clients are scarce. The authors’ qualitative study revealed that sexual harassment is a complex and multidimensional phenomenon. We aim to tap into different aspects of sexual harassment from patients or clients by applying a broad definition of this phenomenon, allowing us to include a wide range of nonverbal (e.g., sexual gestures or acts), verbal (e.g., comments with sexual content), and physical acts (e.g., unwanted physical contact and sexual assaults) of sexual harassment.

### 1.2. The Effect of Sexual Harassment on Employees’ Mental Health

From a conceptual point of view, we argue in line with Barling and colleagues [16] that sexual harassment can be considered a major organizational stressor. A considerable body of research indicates that sexual harassment in the workplace may have detrimental effects on employees’ physical and mental health [6,9,17,18,19]. Empirical studies investigating sexual harassment focused on relationships with indicators of impaired well-being [12,19,20,21]. Fitzgerald, Drasgow, and colleagues [19] showed that sexual harassment is associated with higher levels of depression, and Larsen and Fitzgerald [22] found that sexual harassment is related to posttraumatic stress. Empirical studies indicated that sexual harassment is associated not only with psychological states but also with (psycho)somatic complaints [21,23]. Accordingly, meta-analytical findings provided evidence that victims of sexual harassment report higher levels of depression, general stress, anxiety, posttraumatic stress, and psychosomatic complaints [6,17,18].

Studies on the relationship between sexual harassment and indicators of positive well-being were ambiguous. Although some studies found no significant associations [20,21], meta-analytical findings provided evidence for a negative relationship between sexual harassment and positive well-being (e.g., life satisfaction) [6]. Meta-analytical results also suggested that sexual harassment is negatively related to job satisfaction [6]. 

To date, research focused on sexual harassment from supervisors and colleagues, i.e., intraorganizational sexual harassment. The small number of studies on sexual harassment and sexual violence from clients and customers indicated severe detrimental effects on employees’ health [2,12,13,24]. However, knowledge about the impact of sexual harassment from patients, clients, and customers on employee well-being is limited. Although research suggests that sexual harassment from clients and customers is an important and prevalent issue in occupations that involve interpersonal contact [25,26], empirical studies neglected this extraorganizational perspective on sexual harassment.

A recent cross-occupational study comparing harassment from clients/customers and harassment from other employees among 7603 Danish employees from 1041 organizations found that care workers experienced sexual harassment from clients and customers more often compared to other occupations [12]. Moreover, Friborg and colleagues [12] revealed no difference in levels of depression between care workers who experienced sexual harassment from supervisors and colleagues and care workers who experienced sexual harassment from clients and patients. Both groups reported higher levels of depressive symptoms compared to participants who did not experience sexual harassment [12]. In a study by Barling and colleagues [2], sexual aggression (acts that are deemed offensive but do not involve direct physical act) from clients against homecare workers showed very small positive relationships with indicators of negative mood (anxiety, anger, and sadness), while direct physical acts of sexual harassment showed no relationships with negative mood.

### 1.3. Measurement of Sexual Harassment from an Extraorganizational Perspective 

In the few studies that investigated sexual harassment from patients or clients, the measures for assessing sexual harassment had several weaknesses. Friborg and colleagues [12] assessed the participants’ exposure to sexual harassment from clients or customers using a single-item measure (“Have you been exposed to sexual harassment at your workplace during the last 12 months?”). Research showed that people are reluctant to label sexually inappropriate behavior as sexual harassment [20], and one reason might be that being a victim of sexual harassment is associated with stigmatization and weakness, which may lead to ignorance or trivialization of those experiences [27]. Another reason lies in the nature of healthcare work. According to a qualitative study by Nielsen and colleagues [4], care workers often distinguished between intentional and unintentional sexual behaviors initiated by patients suffering dementia or other cognitive impairments. Because sexual harassment for the participants implied that the actions were intentional, they refrained from using this term. Thus, asking directly if one experienced sexual harassment may result in an underestimation of the occurrence of sexual harassment. Furthermore, we argue that inappropriate sexual behavior may have an impact on care workers’ well-being, even if it is not labeled as sexual harassment. The findings of Magley and colleagues [20] revealed that women who experienced sex-related behaviors reported very similar effects on mental health, irrespective of whether they labeled these experiences as sexual harassment.

In their study of sexual harassment from clients among homecare workers, Barling and colleagues [2] assessed two different aspects of sexual harassment: sexual aggression, i.e., offensive acts that do not involve direct physical acts, and direct physical acts of sexual harassment. Respondents were asked to indicate the frequency of occurrence of the respective event in the last six months. In contrast to the single-item measure used by Friborg and colleagues [12], the items asked about actual events and not about the respondents’ perceptions of sexual harassment. However, a questionnaire with 36 items is relatively lengthy for use in field research. Moreover, the questionnaire remains to be validated.

Bronner, Peretz, and Ehrenfeld [28] developed a questionnaire that assesses sexual harassment in the nursing context, including seven aspects that are each measured with one item (teasing remarks, hearing sex jokes, proposition for an intimate relationship, physical touch, intimate touch, forced to touch intimately, attempt to have sex). However, the authors evaluated only the content validity and reported no information on the psychometric properties of the scale.

To gain an in-depth understanding of the effects of sexual harassment from patients or clients on employee well-being, research needs to use validated measures to assess the full range of sexual harassment acts. Therefore, we followed a multiphase approach to develop a valid and reliable measure of sexual harassment from patients or clients. Firstly, we generated and reviewed potential items that fit our definition of sexual harassment [14,15]. Since our definition is rather broad, we referred to the term inappropriate sexual behaviors, which include a variety of nonverbal, verbal, and physical unwanted sex-related behaviors/acts. We aimed to develop items that reflect observable behaviors instead of asking about sexual harassment directly, as is common practice in single-item measures [12]. We assume that the answer to this direct question depends upon the respondents’ implicit definitions of what constitutes (intentional) sexual harassment and, thus, might result in an underestimation of sexual harassment. In a next step, we examined the factorial structure of the measure using exploratory factor analysis (EFA). Finally, we cross-validated the factorial structure using confirmatory factor analysis (CFA) and evaluated construct validity by investigating relationships with several indicators of mental health. 

The World Health Organization (WHO) defines health as “a state of complete physical, mental, and social well-being and not merely the absence of disease or infirmity” [29]. In line with this definition, many authors acknowledge that well-being includes both (the absence of) negative and positive aspects [30,31]. According to Wright, Emich, and Klotz [32], well-being is a multidimensional construct that comprises indicators of impaired well-being (e.g., indicators of exhaustion) and positive states of mind (e.g., job satisfaction). Previous research indicated that these dimensions are correlated but distinct constructs representing different dimensions rather than two poles on a continuum [33,34]. In this study, we draw on this holistic definition of health, and we assess relationships of sexual harassment with both negative and positive indicators of mental health to obtain a differentiated picture of the impact of sexual harassment. We chose medium-term (strain, emotional exhaustion) and long-term (depression, psychosomatic complaints) indicators of mental health in combination with a relatively long time period in which the experiences occurred (12 months) to rigorously investigate the effects of sexual harassment [35], as well as indicators of positive mental health (positive well-being, job satisfaction).

## 2. Study 1: Item Generation and Item Review

### 2.1. Method

#### 2.1.1. Ethics Approval and Consent to Participate

The research project including all studies received research ethics committee approval from the Local Ethics Committee of the Faculty of Psychology and Human Movement at the Universität Hamburg (no. 2018_195). All participants were informed about the purpose of the study and informed consent was written. 

#### 2.1.2. Sample and Procedure

Based on an extensive literature review, we developed items that assess different forms of sexual harassment from patients or clients in healthcare. Moreover, we conducted a group discussion and interviews with eight experts working in different key positions in healthcare (e.g., equal opportunity commissioners in care for the elderly, nursing, care for the disabled) who endorsed refining the distinction into sexual harassment and sexual aggression and differentiating nonphysical sexual harassment into nonverbal and verbal acts. Nonverbal sexual harassment includes observable sexual gestures or acts. Verbal sexual harassment includes comments with sexual content. Physical sexual harassment refers to the experience of unwanted physical contact and sexual assaults. These behaviors may be perceived as embarrassing, humiliating, intimidating, and/or threatening. We aimed to develop items that assess a broad range of nonverbal, verbal, and physical acts of sexual harassment. For the generation of items, we built heavily upon the measure developed by Barling and colleagues, which is the only high-quality measure we found in the literature that assesses sexual harassment from patients or clients [2]. To complement the item pool, we used measures assessing sexual harassment in other contexts [3,36] and information from the expert interviews. Subsequently, eight healthcare professionals and six researchers reviewed the items in terms of their practical relevance, clarity, and observability.

We included a broad range of behaviors that were identified in the literature and by the eight experts as constituting sexually inappropriate behavior in the professional healthcare context from people outside the organization, such as patients or clients. Consequently, we labeled the measure the Sexually Harassing Behavior Questionnaire from an extraorganizational perspective (SHBQ-X).

### 2.2. Results

This procedure yielded 20 items from the initial item pool, which represented each of the three aspects of sexual harassment with at least four items. Fifteen of these items were transposed and adapted from Barling and colleagues [2]. In the instructions, we emphasized that the items concern experiences of sexual harassment from patients or clients or their relatives in the past 12 months. The inclusion of relatives was a result of the expert discussion. The experts pointed out that relatives play an important role, especially in homecare work, as well as in other institutions. While Barling and colleagues [2] chose a time period of six months, we decided to extend the time period to 12 months because sexual harassment is a rare event and because the experience of sexual harassment may have long-term effects that might not be captured within a shorter time period.

## 3. Study 2: Item Reduction and Factorial Structure

### 3.1. Method

The objective of Study 2 was to reduce the number of items and examine the factorial structure of the measure developed in Study 1.

#### 3.1.1. Sample and Procedure

Numerous healthcare and social service organizations were contacted via email and asked to announce the study in their newsletters, on social media, and on their websites to recruit participants. The sample consisted of 181 employees working in healthcare in Germany. Careless responders were excluded from the analyses when they responded (1) too quickly (i.e., <2 s/item) and (2) the same way to more than 30 items in a row [37]. This approach yielded a final sample size of *N* = 179. Of the participants, 39% worked in hospitals, and 19% were employees working in psychiatric hospitals. In total, 18% of the respondents worked in inpatient care for the elderly, 6% were outpatient caregivers for the elderly, and 7% worked in care for persons with disabilities. The remainder of the participants (11%) worked in other healthcare professions (e.g., forensic psychiatry, medical offices). The participants were aged 19 to 60 years (mean (*M*) = 38.01; standard deviation (*SD*) = 11.56), and 80% were female. On average, the participants worked 34 h per week.

#### 3.1.2. Measures 

*Sexual harassment* was measured using the 20 items that we developed in Study 1. The items were scored on a six-point Likert scale (1 = “never”, 2 = “once”, 3 = “every few months”, 4 = “every few weeks”, 5 = “every few days”, 6 = “(nearly) every day”) during the past 12 months. Table 1 shows the item wordings.

#### 3.1.3. Statistical Analyses

The analyses were performed in R version 3.4.3 (R Foundation for Statistical Computing, Vienna, Austria) [38]. To explore the factorial structure of the measure, we performed EFAs using the covariance matrix. Because we supposed that sexual harassment comprises different aspects (nonverbal, verbal, and physical), we used principal axis factoring which seeks to extract the least number of factors that can account for the common variance. To consider that the different aspects of sexual harassment correlate with each other, we applied oblique (promax) factor rotation.

### 3.2. Results

#### 3.2.1. Item Difficulty

Table 1 shows that four items were negated by more than 85% of the participants. These items were excluded from further analyses because item difficulty was considered too low.

#### 3.2.2. Exploratory Factor Analysis (EFA)

Several methods were combined to determine the number of factors to retain, including the Kaiser–Guttman eigenvalue-greater-than-one rule, the scree plot, and parallel analysis. We performed parallel analysis calculating eigenvalues from 1000 samples of random data and using the 95th percentile of eigenvalues for comparison purposes. These methods suggested retaining three factors. Table 1 shows the factor structure, factor loadings, communalities, eigenvalues, and percentage of variance accounted for.

Factor 1 consisted of five nonverbal sexual harassment items, Factor 2 consisted of six verbal sexual harassment items, and Factor 3 consisted of five physical sexual harassment items. Two items (nonverbal 3 and physical 1) were deleted because they cross-loaded on multiple factors. After excluding these items, we performed a second EFA using the final set of 14 items. Standardized factor loadings ranged from 0.57 to 0.84. The three factors explained 69.1% of the variance. The correlation between Factor 1 and Factor 2 was *r* = 0.59, the correlation between Factor 1 and Factor 3 was *r* = 0.64, and the correlation between Factor 2 and Factor 3 was *r* = 0.46. Table 2 displays the means, standard deviations, and internal consistencies of the scales. The scales demonstrated acceptable internal consistencies.

## 4. Study 3: Validation of the Factorial Structure and Relationships with Employee Mental Health

### 4.1. Method

To validate the factorial structure revealed in Study 2, we conducted a CFA. Moreover, we examined relationships between sexual harassment and several indicators of employee mental health using correlations to evaluate construct validity.

#### 4.1.1. Sample and Procedure

To obtain a heterogeneous sample, we collected data from 320 healthcare workers in Germany in cooperation with a market research institution. Careless responders were identified through response time and longstring index (see Study 1). The final sample size was *N* = 305 with a mean age of 43.5 years (*SD* = 10.7) and *M* = 31.8 working hours per week (*SD* = 10.2). In total, 79% of the participants were female; 31% of the sample were employees working in hospitals, 16% worked in inpatient care for the elderly, 11% worked in in outpatient care for the elderly, 10% worked in psychiatric clinics, and 9% worked in forensic psychiatry. The remainder of the participants (18%) worked in other healthcare occupations (e.g., dental office, medical office, physiotherapy).

#### 4.1.2. Measures

Sexual harassment was assessed using the final set of 14 items validated in Study 2, and 10 of these items were transposed and adapted from Barling and colleagues [2]. 

Emotional exhaustion was measured using the nine-item subscale from the Maslach Burnout Inventory (MBI) [39] in its German version [40]. Emotional exhaustion is at the core of burnout and reflects feelings of being overextended and depleted of emotional and physical resources [41]. A sample item is “I feel drained from my work.” Items were scored on a five-point Likert scale ranging from 1 (“a few times a year or less frequently”) to 6 (“every day”). 

To assess depressive symptoms, we used six items from the Center for Epidemiological Studies Depression Scale (CES-D) [42] in its German translation [43]. Viewing depression as a continuous construct, this measure assesses depressive symptoms in the general population by asking respondents for their experience of several depressive symptoms. The measure focuses on the affective component, depressed mood. Although not a clinical assessment, high scores may be interpreted as being at risk of depression. A sample item is “Over the last two weeks, I felt depressed.” Items were scored on a seven-point Likert scale ranging from 1 (“not at all”) to 7 (“nearly every day”). 

Psychological strain was assessed with three items measuring emotional irritation [44]. Psychological strain refers to a state of heightened physiological arousal and emotional irritability, which manifests in one’s affective experience (e.g., being grumpy) and behavior (e.g., getting angry easily). A sample item is “I get irritated easily although I do not want this to happen.” Items were scored on a seven-point scale ranging from 1 (“strongly disagree”) to 7 (“strongly agree”). 

Psychosomatic complaints were assessed using ten items [45], and a sample item is “Do you have headaches?” To measure positive well-being, we used the WHO-5 well-being index [46,47]. A sample item is “Over the last two weeks, I have felt calm and relaxed.” Items were scored on a six-point Likert scale ranging from 1 (“at no time”) to 6 (“all the time”). 

Job satisfaction was assessed using seven items from the Copenhagen Psychosocial Questionnaire (COPSOQ) [48] in its German version [49]. A sample item is “Regarding your work in general, how pleased are you with your work prospects?” Items were scored on a five-point Likert scale ranging from 1 (“very unsatisfied”) to 5 (“highly satisfied”).

#### 4.1.3. Statistical Analyses

The analyses were performed in R version 3.4.3 [38]. To test the hypothesized three-factor structure of the measure, CFA using maximum-likelihood estimation was conducted using the lavaan package [50]. In contrast to EFA, which was used to explore the factorial structure of the measure, CFA allows for the assessment of an a priori conceptualized measurement model. As an indicator of the overall fit of the models, we computed chi-square statistics. Nonsignificant chi-square values indicate that the model fits the data well. Because chi-square statistics are sensitive to sample size [51], additional fit indices were considered: the comparative fit index (CFI), squared root mean residual (SRMR), and root-mean-square error of approximation (RMSEA). General guidelines suggest that values close to 0.95 or higher for CFI, levels of 0.08 or lower for SRMR, and levels of 0.06 or lower for RMSEA indicate adequate fit [52]. To compensate for deviations from multivariate normality, Satorra–Bentler-scaled test statistics and robust standard errors were computed. 

### 4.2. Results

#### 4.2.1. Confirmatory Factor Analysis (CFA) 

**Figure 1:**
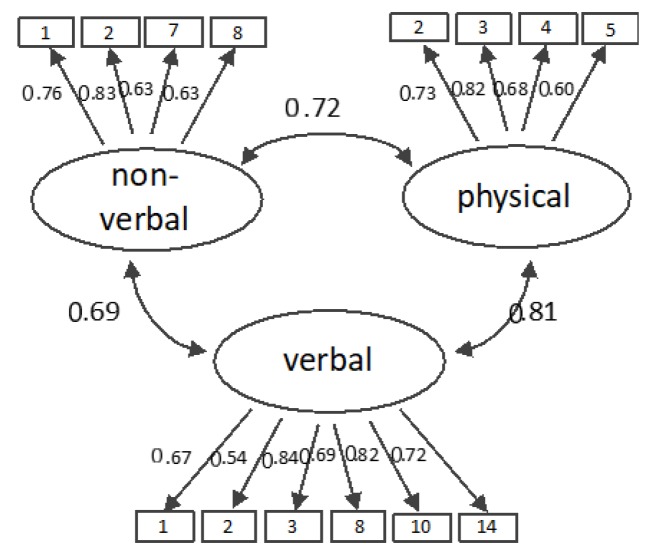
Standardized factor loadings and correlations between the latent factors in the three-factor model.

Firstly, we tested the three-factor model revealed in Study 2 in which all indicators loaded onto their respective factors. The latent factors were allowed to covary. For the three-factor model, the results indicated an adequate fit (χ^2^(74) = 104.75, *p* = 0.011; CFI = 0.97; RMSEA = 0.037; SRMR = 0.047). Figure 1 shows the standardized loadings and correlations between the latent factors. Secondly, we specified an alternative measurement model to assess whether nonverbal and verbal sexual harassment were distinct factors. In this two-factor model, the indicators of nonverbal and verbal sexual harassment loaded onto one latent factor, and the indicators of physical sexual harassment loaded onto another latent factor. This two-factor model achieved poor fit with the data (χ^2^(76) = 181.58, *p* < 0.001; CFI = 0.88; RMSEA = 0.067; SRMR = 0.071). Finally, we tested a one-factor model in which all items loaded on one latent factor. This model also yielded poor fit with the data (χ^2^(77) = 202.07, *p* < 0.001; CFI = 0.86; RMSEA = 0.073; SRMR = 0.073). Thus, the CFA provides evidence that the different aspects of sexual harassment represent three related yet distinct factors.

#### 4.2.2. Correlations between Sexual Harassment and Employee Well-Being

Table 3 shows the correlations between sexual harassment and care workers’ well-being. Nonverbal sexual harassment was positively related to emotional exhaustion, psychological strain, depressive symptoms, and psychosomatic complaints and negatively related to positive well-being and job satisfaction. The correlations between verbal sexual harassment and care workers’ impaired well-being were also positive. Moreover, verbal sexual harassment was negatively related to positive well-being but was not significantly related to job satisfaction. Physical sexual harassment was positively related to impaired well-being (i.e., emotional exhaustion, psychological strain, depressive symptoms, and psychosomatic complaints) and negatively related to positive well-being and job satisfaction.

## 5. Discussion

It is widely regarded that care workers often encounter sexual harassment from patients or clients and that these experiences have detrimental effects on employees’ mental health [1,12]. Although sexual harassment from clients and customers is considered an important and prevalent issue, especially in occupations that involve interpersonal contact [25], theoretical developments are weak, and validated measures that may help gain in-depth insights into this phenomenon are missing. Therefore, the aim of this study was to develop and validate a measure for assessing different types of sexual harassment from patients or clients in the healthcare sector. As a conceptual foundation for this measure, we introduced a definition of sexual harassment based on three different types, nonverbal, verbal, and physical sexual harassment, in line with the definition by the European Commission [15].

The findings show that the SHBQ-X is a valid measure for assessing the impact of sexual harassment from clients or patients on employees’ mental health. CFAs provide evidence that the different aspects of sexual harassment represent three related yet distinct factors. The results give credit to the notion that sexual harassment is not a unidimensional construct but rather multifaceted, comprising nonverbal, verbal, or physical acts. Furthermore, these factors show substantial relationships with various indicators of employees’ well-being. These relationships are in line with former studies that show detrimental effects of sexual harassment by patients or clients on employees’ mental health and job satisfaction [1,6]. Whereas physical sexual harassment shows similar correlations with positive and negative well-being indicators, nonverbal and verbal sexual harassment are more strongly related to indicators of impaired well-being than to job satisfaction. These results suggest that nonverbal and verbal sexual harassment might be more relevant for diseases such as burnout or depression and have less of an effect on job satisfaction. As stated, we consider sexual harassment to be a major organizational stressor. According to Semmer and colleagues [53], correlations between stressors and strain are typically between *r* = 0.20 and *r* = 0.30. Our results show similar correlations for sexual harassment, such as other job characteristics (e.g., workload, role conflict), which underlines the assumption that sexual harassment can be considered a major organizational stressor.

Unlike other studies [2], in which the researchers chose a time frame of six months during which the acts of sexual harassment occurred, we expanded the time frame to 12 months. The fact that we found correlations >0.20 with multiple indicators of employees’ mental health emphasizes the heath relevance of sexual harassment and may indicate that the health-related effects of sexual harassment may be quite stable or develop over time.

The substantial relationships with depression and psychosomatic complaints give credit to the notion that sexual harassment might have a stronger impact on medium- and long-term indicators of employee well-being than on indicators with a shorter reaction time, e.g., negative mood, which showed much lower correlations in Barling and colleagues’ [2] study with homecare workers.

We are aware that care workers are reluctant to label inappropriate sexual behavior from patients as sexual harassment, especially if the patients are demented or cognitively impaired and, therefore, not aware of their actions [4]. In addition, it is likely that individuals rate the same behaviors differently. While some people may consider a behavior sexually harassing, others may not share this view. Therefore, we refrained from asking the respondents whether the sexual acts were perceived as sexual harassment. Rather, we assumed that the experienced behaviors assessed by the measure have an impact on well-being, regardless of whether healthcare workers actually label the patients’ or clients’ behaviors as sexual harassment. The finding that the measure showed substantial correlations with indicators of healthcare workers’ well-being provides support for this notion. 

### 5.1. Future Directions

Although it is widely regarded that multiple factors determine sexual harassment, research lacks an integrative definition of and theoretical framework for sexual harassment. As pointed out by Quick and McFadyen [1] in their review, much more work is needed to better understand sexual harassment in the workplace and ways in which to decrease its occurrence. In line with the premise that sexual harassment is a function of organizational and job characteristics [19], we consider sexual harassment a preventable occupational health problem. Hence, the identification of risk factors in the occurrence of sexual harassment or protective factors in coping with sexual harassment (from patients or clients) is an important task for future research.

Traditionally, organizational psychology plays a prominent role in sexual harassment research investigating how organizational factors such as power and inequality, policies, and organizational climate determine the occurrence of sexually harassing behaviors [4,17]. Organizational climate was found to be the strongest empirical forecaster of sexual harassment from supervisors and colleagues in the workplace [6,54]. Meta-analytical findings provided support for the notion that organizational climate in terms of organizations’ intolerance for sexual harassment and explicit norms and policies is a key factor in determining the occurrence and management of sexual harassment [1,4,6].

A direction for future research is to develop a unifying theoretical framework for sexual harassment that encompasses antecedents, consequences, and possible mediator or moderator variables to explain the dynamic process of sexual harassment. The job demands–resources (JD-R) model [55] offers a suitable theoretical basis for a holistic conceptual framework. We consider sexual harassment a job demand that can lead to employees’ impaired well-being (e.g., burnout, depression). Building on the JD-R model, we postulate that a number of organizational resources (e.g., social support, organizational climate) or personal resources (e.g., hardiness, self-efficacy) can buffer the relationship between this demand and impaired well-being. Expanding the JD-R model, one should also examine potential risk factors for the occurrence of sexual harassment. A promising approach is to integrate organizational risk factors that may increase the likelihood of the occurrence of sexual harassment (e.g., power distance, job gender context, organizational climate, patient–staff ratio) and personal risk factors of the employee (e.g., neuroticism, low self-esteem) or the patient/client (e.g., specific diseases, such as dementia, personal characteristics, such as gender and age, and disorders, such as hostility and narcissism), as it is argued that causal explanatory theories of sexual harassment should not consider only organizational aspects [54]. A multilevel study incorporating an organizational and individual perspective could be a promising approach.

While most studies examined intraorganizational sexual harassment from colleagues or supervisors, we aimed to shed light on an extraorganizational perspective, including clients and patients. For future studies, we recommend integrating different perspectives into one study to compare the impact of the different sources of harasser.

Although we found substantial correlations between the different sexually harassing behaviors and the indicators of employee well-being, it is conceivable that the interpretation of the behavior as an intentional offensive act of harm may strengthen the relationship between the experience of sexual harassing behaviors from patients or clients and employee well-being. On the other hand, healthcare workers’ perception that sexually harassing behaviors are due to patients’ disease pattern (e.g., dementia) might attenuate the relationship between sexually harassing behaviors and well-being. Therefore, future research may benefit from considering healthcare workers’ attributions of sexually harassing behavior to advance the understanding of moderators of the relationship between sexually harassing behaviors and well-being.

### 5.2. Practical Implications

Sexual harassment is a socially sensitive topic. The experience of patients’ sexual needs is a frequently occurring part of care work; therefore, it is difficult for care workers to label sexually inappropriate behavior as sexual harassment [4]. As a result, in healthcare, there is a tendency to trivialize sexual harassment from patients or clients as a part of the job and not as a potentially harmful experience [25,56]. Subsequently, a reason for the limited interest in sexual harassment from patients or clients may be that organizations may normalize and neglect the seriousness of this act [12,25]. A problem in person-related professions is the lack of definition of what kinds of behavior are to be accepted, which leads to difficulties in distinguishing between inappropriate sexual behavior from clients and work-related responsibilities [12].

A culture of high tolerance for and denial of sexual harassment in occupations that involve interpersonal contact may increase the likelihood of the occurrence of sexual harassment and may affect its detrimental impact on employees’ mental health. Nielsen and colleagues [4] criticized that workplaces rarely have special guidelines or policies defining inappropriate sexual behavior and that incidents are often managed in an ad hoc and individualized manner. There is a need to put sexual harassment on the agenda and to develop standard operational procedures regarding the management of sexual harassment to prevent and mitigate negative effects. As demographics change with the rising number of elderly and the increase in various forms of dementia on the one hand and skills shortages in the healthcare sector on the other hand, the prevention and management of inappropriate sexual behavior by patients will be an even more pressing issue for organizations in the future that seek to ensure that employees are healthy and safe and can work efficiently on a long-term basis. 

To keep healthcare workers safe and healthy, it is important to provide knowledge about prevalence rates and various forms of sexual harassment to remove taboos and develop appropriate prevention and rehabilitation strategies. This knowledge should be integrated into prevention and rehabilitation programs. The prevalence of sexual harassment from patients or clients differs considerably because, until now, we did not have a commonly accepted measure for the valid assessment of sexual harassment. With the SHBQ-X, we can now provide reliable prevalence rates in the future to estimate the practical relevance of this problem.

New training elements are encouraged since exposures to sexual harassment and violence are not common elements in the education of healthcare workers. Leaders may play an important role in fostering an organizational culture in which employees believe that the organization will take complaints seriously. The focus of leadership interventions that aim to decrease the prevalence of sexual harassment and its detrimental impact on employee well-being should provide leaders with practical strategies and tools for shaping employees’ work environment.

However, we also recommend implementing organizational-level interventions [57]. Leaders who receive support from the organization are in a better position to benefit their employees [58,59]. Thus, organizations should provide leaders with adequate support (e.g., though the establishment of preventive guidelines and procedures) to allow them to promote their employees’ well-being through the prevention or more adequate management of sexual harassment.

### 5.3. Limitations 

When interpreting the findings, some limitations should be considered. Firstly, the study used cross-sectional data, which restrict any conclusions about the causality of the effects. Thus, reverse causal effects of employee health on the perception of sexual harassment are possible [60]. For example, employees with low levels of mental health may be more likely to perceive patients’ or clients’ behavior as sexually inappropriate. Furthermore, employees with impaired mental health may have a higher risk of actually experiencing sexual harassment because they might be an easy target for perpetrators. Therefore, we suggest that future studies examine the associations between the SHBQ-X and employee well-being using longitudinal designs.

Moreover, the analyses used single-source and single-method data; the participants assessed both their experience of sexually inappropriate behavior and their well-being. Therefore, common method variance may have inflated the relationships [61]. Future research should take this limitation into account and use multiple sources of information and mixed methods approaches (e.g., colleagues, supervisors, clients), different assessment methods (e.g., surveys, interviews, observations), different study designs (e.g., multilevel design), and multiple indicators of employee well-being (e.g., self-reports and behavioral and physiological measures) [62] to examine the impact of sexual harassment on employees’ mental health.

Finally, because the results show that nonverbal and verbal sexual harassment are significantly correlated, one might question whether these two types of sexual harassment represent two distinct constructs. However, the CFA revealed that the three-factor model fit the data better than the two-factor model. 

## 6. Conclusions

Based on the findings for content and construct validity, we suggest that the SHBQ-X is a useful and valid tool to examine sexual harassment from patients or clients. In contrast to single-item measures asking for perceptions of sexual harassment, the SHBQ-X allows for the definition of specific and observable behaviors/acts of sexual harassment that can impact care workers’ mental health. Our findings advance the understanding of how patients or clients may affect employees’ mental health through sexually inappropriate nonverbal, verbal, or physical behaviors. When conducting field research, survey space is at a premium, and having a comprehensive, psychometrically sound measure is critical. We suggest that our measure will be beneficial to researchers who are seeking to assess different types of sexual harassment by patients or clients in healthcare or to develop and test a theoretical framework for the antecedents and consequences of sexual harassment. This measure combines scientific and practical value, and it provides a contribution to preventive and prospective health promotion in healthcare.

## Figures and Tables

**Table 1:** Exploratory factor analysis of the initial and final set of sexual harassment items.

Title			Initial Set of 16 Items				Final Set of 14 Items			
Item	Item Wording	% “never”	1	2	3	*h* ^2^	1	2	3	*h* ^2^
**Nonverbal 1**	I have witnessed sexual acts (e.g., masturbation).	48.0			**0.80**	0.69		**0.81**		0.69
**Nonverbal 2**	I have witnessed sexual gestures.	32.4			**0.69**	0.71		**0.69**		0.69
Nonverbal 3	I have been leered/stared at.	33.0	0.52	0.32		0.63				
**Nonverbal 4**	Someone has unnecessarily exposed themselves in front of me.	67.6			**0.68**	0.59		**0.67**		0.57
**Nonverbal 5**	I have witnessed sexual harassment/violence among patients/clients/residents.	65.4			**0.79**	0.56		**0.78**		0.57
**Verbal 1**	I have been whistled at.	49.2	**0.77**			0.55	**0.71**			0.53
**Verbal 2**	I have received repeated requests for dates.	75.4	**0.62**			0.45	**0.61**			0.46
**Verbal 3**	I have been sexually complimented.	34.6	**0.88**			0.74	**0.84**			0.73
**Verbal 4**	I have been told suggestive/offensive stories or jokes.	35.2	**0.56**			0.50	**0.57**			0.52
**Verbal 5**	I have been exposed to verbal sexual innuendo.	42.5	**0.63**			0.70	**0.62**			0.71
**Verbal 6**	I have been asked intrusive or personal questions by a client (e.g., requests for body measurements, relationship status, sexual preferences).	62.0	**0.66**			0.55	**0.68**			0.59
Physical 1	I have experienced someone unnecessarily close/breaking personal boundaries.	34.1	0.43	0.36		0.57				
**Physical 2**	I have been hugged in a way that made me feel uncomfortable.	65.4		**0.76**		0.66			**0.72**	0.63
**Physical 3**	I have been petted or patted in a way that made me feel uncomfortable.	63.7		**0.85**		0.77			**0.84**	0.75
**Physical 4**	I have been touched in a way that made me feel uncomfortable.	71.0		**0.75**		0.62			**0.76**	0.64
**Physical 5**	I have been kissed in a way that made me feel uncomfortable.	82.1		**0.67**		0.40			**0.67**	0.43
Eigenvalue			7.98	1.73	1.14		6.85	1.69	1.13	
Variance accounted for (%)			49.90	10.84	7.11		48.92	12.06	8.08	
Verbal 7	I have been targeted for rumors of sexual promiscuity.	**87.7**								
Verbal 8	I have been exposed to insults that targeted my sexual orientation.	**90.5**								
Verbal 9	I have been offered money for sex.	**92.7**								
Physical 6	I have been cornered or placed in a position that was difficult to get out of.	**95.0**								

Note: *N* = 179. Standardized factor loadings ≥0.55 are in boldface; *h*^2^ = communalities.

**Table 2:** Means (*M*), standard deviations (*SD*), and internal consistencies of the calibration sample.

Scale	*M*	*SD*	α
Nonverbal sexual harassment	1.95	1.04	0.86
Verbal sexual harassment	2.04	1.02	0.89
Physical sexual harassment	1.49	0.78	0.85

Note: *N* = 179. α = Cronbach’s alpha.

**Table 3:** Descriptive statistics, bivariate correlations, and Cronbach’s alphas.

Variables	*M*	*SD*	1	2	3	4	5	6	7	8	9
1 Nonverbal SH	1.45	0.69	(0.79)								
2 Verbal SH	1.69	0.83	0.57 ***	(0.86)							
3 Physical SH	1.38	0.65	0.60 ***	0.67 ***	(0.80)						
4 Emotional exhaustion	2.98	1.29	0.21 ***	0.21 ***	0.26 ***	(0.94)					
5 Strain	2.61	1.27	0.16 **	0.13 *	0.18 **	0.53 ***	(0.89)				
6 Depressive symptoms	2.38	1.30	0.27 ***	0.26 ***	0.27 ***	0.64 ***	0.53 ***	(0.94)			
7 Psychosomatic complaints	2.32	0.83	0.25 ***	0.27 ***	0.25 ***	0.67 ***	0.46 ***	0.74 ***	(0.88)		
8 Positive well-being	3.26	0.89	−0.24 ***	−0.21 ***	−0.28 ***	−0.56 ***	−0.46 ***	−0.64 ***	−0.63 ***	(0.92)	
9 Job satisfaction	3.55	0.87	−0.15*	−0.09	−0.24 ***	−0.56 ***	−0.35 ***	−0.49 ***	−0.47 ***	0.54 ***	(0.91)

Note: *N* = 305. Cronbach’s alphas in parentheses on the diagonal. * *p* < 0.05, ** *p* < 0.01, *** *p* < 0.001. SH: sexual harassment.

## Data Availability

The data are available from the authors upon request.
